# Probiotics and Atopic Dermatitis: An Overview

**DOI:** 10.3389/fmicb.2016.00507

**Published:** 2016-04-12

**Authors:** Irfan A. Rather, Vivek K. Bajpai, Sanjay Kumar, Jeongheui Lim, Woon K. Paek, Yong-Ha Park

**Affiliations:** ^1^Department of Applied Microbiology and Biotechnology, School of Biotechnology, Yeungnam UniversityGyeongsan, South Korea; ^2^Department of Clinical Studies, College of Veterinary Medicine, New Bolton Center University of Pennsylvania, Pennsylvania, PAUSA; ^3^National Science Museum, Ministry of Science, ICT and Future Planning, DaejeonSouth Korea

**Keywords:** atopic dermatitis, skin diseases, inflammation, clinical trials, probiotics

## Abstract

Atopic dermatitis (AD) is a common, recurrent, chronic inflammatory skin disease that is a cause of considerable economic and social burden. Its prevalence varies substantially among different countries with an incidence rate proclaimed to reach up to 20% of children in developed countries and continues to escalate in developing nations. This increased rate of incidence has changed the focus of research on AD toward epidemiology, prevention, and treatment. The effects of probiotics in the prevention and treatment of AD remain elusive. However, evidence from different research groups show that probiotics could have positive effect on AD treatment, if any, that depend on multiple factors, such as specific probiotic strains, time of administration (onset time), duration of exposure, and dosage. However, till date we still lack strong evidence to advocate the use of probiotics in the treatment of AD, and questions remain to be answered considering its clinical use in future. Based on updated information, the processes that facilitate the development of AD and the topic of the administration of probiotics are addressed in this review.

## Introduction

Atopic Dermatitis (AD), also known as eczema, is a chronic inflammatory, relapsing, and non-contagious skin disease that is known to affect ≈20% of children in both developed and developing countries (Shaw et al., 2011; Deckers et al., 2012). AD represents one’s first allergic reaction encountered in childhood and is recognized as a precursor for the development of a series of hypersensitivity reactions such as food allergies, asthma, and allergic rhinitis (Castro-Rodriguez, 2010; Finnbogadóttir et al., 2012; Carlsten et al., 2013; Silverberg and Simpson, 2013). Classical treatment guidelines for AD include hydrating topical treatment, topical glucocorticosteroids (Schmitt et al., 2010), topical calcineurin inhibitors, and concurrent adjuvant therapy, which includes UV radiations (UVA1 and UVB 311 nm) (Williams and Grindlay, 2008; Gambichler et al., 2009; Majoie et al., 2009). Traditional therapeutic strategies have been efficacious in ameliorating the symptoms of AD in most patients (Lio et al., 2012). However, a more comprehensive and mechanistic understanding of the underlying immunological processes is needed to instigate the development of novel applicable treatment approaches for AD.

## Genetic Predisposition to AD and Underlying Immunopathological Process

Atopic dermatitis is a skin disease that is characterized by compromised skin barrier integrity, heightened inflammatory response against stimulants, and diminished antimicrobial responses that incite abnormal inflammation in the skin. The underlying mechanism and etiology of AD remain unexplored. AD is a complex skin problem caused by an interplay between genetic susceptibility and prenatal/postnatal environmental factors (Williams and Flohr, 2006). Genome wide screens have linked AD to several chromosomal loci, including 3q21, 5q31, and 11q13 (Lee et al., 2000; Bowcock and Cookson, 2004; Haagerup et al., 2004; Hoffjan and Epplen, 2005). The candidate genes interestingly encode immunomodulators and co-stimulatory proteins involved in T-cell activation, as well as cytokines involved in the regulation of IgE synthesis, such as interleukin-3, interleukin-4, interleukin-5, interleukin-11, and the granulocyte–macrophage colony-stimulating factor (GM-CSF) (Fölster-Holst et al., 1998; Kawashima et al., 1998; Forrest et al., 1999; Leung and Bieber, 2003). A study identified loss-of-function non-sense mutations in the filaggrin gene (FLG) that is associated with AD (Marenholz et al., 2006; Palmer et al., 2006; Sandilands et al., 2007; Margolis et al., 2013). The FLG encodes a protein that is responsible for retaining moisture and protecting the skin from environmental allergens; therefore, it is crucial for maintaining skin barrier integrity.

Prenatal and postnatal maternal diet, gestational diabetes, exposure to microorganisms, and allergens are potential risk factors associated with the onset and development of AD (Cipriani et al., 2014). Several epidemiological and experimental evidences support the theory of “hygeine hypothesis” as the most reasonable explanation for the AD epidemics in last few decades (Martinez, 2001). The hygeine hypothesis suggests that changes in immunoregulatory infectious environment and the patterns of microbial exposure of children that are associated with Westernized culture are critical factors underlying the increasing severity and prevalence of atopic disorders. A study conducted by Strachan (1989) demonstrated an inverse correlation between sibship size and the subsequent risk of allergy, and it was recently confirmed by a broad international study involving more than 500,000 children in 52 countries (Strachan et al., 2015). Briefly, hygiene hypothesis inversely relates the prevalence of allergic diseases and urban lifestyles, high standard sanitary conditions, vaccinations, antibiotic administration, and small family size. Westernized lifestyle scales down infantile exposure to the allergens, which translates into decreased Th1-driven immune responses and favors less mature neonatal Th2-mediated immune systems, which may be the cause of the onset of allergic diseases (Strachan, 2000).

## Probiotics and Prevention of Atopic Dermatitis

Probiotics are live microorganisms that, when administered in sufficient amounts, confer health benefits on the host (Hill et al., 2014). They barricade the epithelium and mucosal surfaces in the intestine, thereby preventing the adherence and invasion of pathogens (Servin and Coconnier, 2003). After birth the host receives primary microbial stimulus through the installation of gut microbiota or through exposure to specific bacterial strains. The establishment of microbial flora in the early postnatal period activates the innate and adaptive immune system, and the uninterrupted microbial stimulus serves to mature the gut mucosal immune system. Early compromised microbial stimulus may lead to reduced intestinal surface area, incoordination and alteration in the mucosal intermediary metabolism, a sensitive mucosal barrier, and a secretory mucosal IgA system (Gaskins, 1997; Cebra, 1999). An imbalance in Th1/ Th2 immune response has been related to the pathogenesis of allergic diseases (Romagnani et al., 1991; Romagnani, 2000; Schmidt-Weber and Blaser, 2004). Probiotics contribute to regulating allergic hypersensitivity reactions by suppressing the Th2 mediated response that helps in balancing Th1/ Th2 immune responses and by increasing Treg mediated immune responses (Feleszko et al., 2007; Kim et al., 2012; Kim J.Y. et al., 2013).

A large number of studies have explored the potential efficacy of probiotics in the prevention and treatment of AD (Pessi et al., 2000; Kalliomaki et al., 2001; Ouwehand et al., 2002; Hattori et al., 2003; Matsumoto et al., 2007; Park et al., 2008; Savilahti et al., 2008; Wickens et al., 2008; Adams, 2010; Batchelor et al., 2010; Chapman et al., 2011; Wickens et al., 2012; Morgan et al., 2014), yet the picture remains unclear and conflicting (**Table 1**). *Lactobacillus rhamnosus* GG (LGG) is the most frequently studied probiotic strain. AD prevention studies have been carried out on children at high risk of AD, and probiotic administration was done 2–4 weeks prenatally to the pregnant mothers and postnatally to the infants for a 1-year time period (Frei et al., 2015). The epidemiological study of a cohort from Norway investigated the potential association between the administration of probiotic milk during pregnancy and infancy period and the onset or establishment of atopic diseases such as AD, rhinoconjunctivitis, and asthama. This study demonstrated an inverse correlation between the intake of probiotic milk products and the incidence of AD; however, the certainty of the evidence is low (Bertelsen et al., 2014). Another study evaluated the impact of *Bifidobacterium breve* M-16V and *Bifidobacterium longum* BB536 administration over the time period of 1 month prenatally, 6 months during infancy, and a period of 18 months follow up on the management of allergic diseases (Enomoto et al., 2014). The study concluded that the incidence of AD was lower in the probiotic administered cases than the controls. A study performed by Rautava et al. (2012) investigated the preventive effects of *L. rhamnosus* LPR, *B. longum* BL999, and *L. paracasei* ST11, during 2 months before and after the expected date of delivery. They reported less episodes of AD in the infants of mothers who received any of the probiotic supplements compared to the placebo group; however, there was no difference in skin prick tests among the experimental groups (Rautava et al., 2012). A number of studies on LGG suggest that the combination of probiotic strains and prebiotic mixtures imposes positive effects in terms of preventing the onset of AD (Kukkonen et al., 2007; Mikael, 2013; Foolad and Armstrong, 2014). However, strong evidence to support the effectiveness of the administration of probiotics at a clinical level remains elusive (Meninghin et al., 2012; Foolad and Armstrong, 2014).

**Table 1:** Effect of probiotics (single or mixed culture) on treatment of Atopic Dermatitis (AD) in humans.

Reference	Probiotics	Outcome
Majamaa and Isolauri, 1997	*Lactobacillus rhamnosus strain GG*	SCORAD score improvement (*P* = 0.008)
Rosenfeldt et al., 2003	*L. rhamnosus* + *L. reuteri*	Positive effect of probiotics seen in allergic subjects (*P* = 0.04)
Kirjavainen et al., 2003	*L. rhamnosus strain GG or L. GG*	SCORAD decrease (*P* = 0.02)
Viljanen et al., 2005	*L. rhamnosus strain GG*	Positive effect seen only in IgE-sensitized infants (*P* = 0.036)
Weston et al., 2005	*L. fermentum*	SCORAD decrease (*P* = 0.03)
Passeron et al., 2006	*L. rhamnosus*/synbiotics	No significant difference between synbiotics and placebo
Sistek et al., 2006	*L. rhamnosus* + *B. lactis*	Positive effect seen only in food-sensitized children (*P* = 0.047)
Brouwer et al., 2006	*L. rhamnosus strain GG or L. rhamnosus*	No significant difference between probiotics and placebo
Fölster-Holst et al., 2006	*L. rhamnosus strain GG*	No significant difference between probiotics and placebo
Grüber et al., 2007	*L. rhamnosus strain GG*	No significant difference between probiotics and placebo
Roessler et al., 2008	*L. paracasei* + *L. acidophilus* + *B. lactis*	No significant effects of probiotics
Chernyshov, 2009	*L. rhamnosus* 95%, *L. helveticus* 5%	SCORAD decrease in subjects not used topical steroids was shown only in probiotic group (*P* < 0.01)
Gerasimov et al., 2010	*L. acidophilus* + *B. lactis*	SCORAD decrease (*P* = 0.001)
Woo et al., 2010	*L. sakei*	SCORAD decrease (*P* = 0.008)
Cukrowska et al., 2010	*L. casei* + *L. paracasei*	Clinical improvement seen mostly in children with IgE-dependent atopic eczema
Gobel et al., 2010	*L. acidophilus*, or *B. lactis*/—	No beneficial effects observed
van der Aa et al., 2010	*B. breve/*synbiotics	Improvement in IgE-sensitized infants (*P* = 0.04)
Yoshida et al., 2010	*B. breve*	SCORAD decrease (*P* = 0.034)
Wu et al., 2011	*L. salivarius/ / synbiotics*	SCORAD decrease at 8 week (*P* = 0.022)
Farid et al., 2011	*mixture/synbiotics*	SCORAD decrease (*P* = 0.001)
Gore et al., 2012	*L. paracasei or Bifidobacterium*	No significant difference between probiotics and placebo
Yesilova et al., 2012	*Bifidobacterium bifidum* + *L. acidophilus* + *L. casei* + *L. salivarius*	SCORAD decrease (*P* = 0.0015)
Han et al., 2012	*L. plantarum*	SCORAD decrease (*P* = 0.0015)
Iemoli et al., 2012	*L. salivarius; + B. breve*	SCORAD decrease (*P* < 0.001)
Drago et al., 2011	*L. salivarius*	SCORAD decrease (*P* < 0.001)
Niccoli et al., 2014	*L. salivarius* LS01	SCORAD and itch improvement

There are studies that state that the use of probiotics is ineffective in the management of AD. The incidence of AD was investigated when the infants who had received probiotic strains of *L. salivarius* CUL61, *L. paracasei* CUL08, *Bifidobacterium animalis* subspecies lactis CUL34, and *Bifidobacterium bifidum* CUL20 reached 2 years of age, and the results were compared to those of toddlers who had not received the placebo. The administration of probiotics did not intervene in the development of AD (Allen et al., 2014).

## Treatment of Atopic Dermatitis

Evidence supporting the use of probiotics for the treatment and prevention of AD is very limited. A restricted amount of evidence suggests that probiotics can decrease the severity of AD. A randomized, double-blind, placebo-controlled study investigated the effects of the use of the *L. plantarum* CJLP133 strain in the prevention of AD symptoms. The study was performed for a time period of 12 weeks among children who were one and 12 years old. It was found that there was an improvement in AD scores (SCORAD), with a concomitant decrease in IFN-γ, eosinophil, and Interleukin-4 counts (Han et al., 2012). Another randomized, double-blind, placebo-controlled study investigated the use of *L. paracasei* (LP), *L. fermentum* (LF), and LP+LF together in children, and it was observed that the SCORAD scores were lower in the group that received probiotics than those of the placebo group 4 months after discontinuing the probiotic treatment (Wang and Wang, 2015). Woo et al. (2010) compared children who received *L. sakei* supplementation to those who received a placebo in a double-blind, placebo-controlled trial. It was found that the supplementation of *L. sakei* was associated with substantial clinical improvement with concomitant decrease in chemokine levels (Woo et al., 2010). Previous meta-analyses evaluating the effects of probiotics on the treatment of AD have resulted in inconsistent results (Boyle et al., 2008; Lee et al., 2008; Michail et al., 2008; Kim et al., 2014). Of these meta-analyses, the most recent one concluded that probiotics significantly improved the SCORAD index in patients aged 1 year or older with AD (mean difference, -4.51; [95% CI, -6.78 to -2.24]; Kim et al., 2014), but the clinical significance of these findings has been questioned, and therefore, the role of probiotics in the treatment of AD has not been definitively established.

## Mechanistic Insights Into Functioning of Probiotics

**FIGURE 1:**
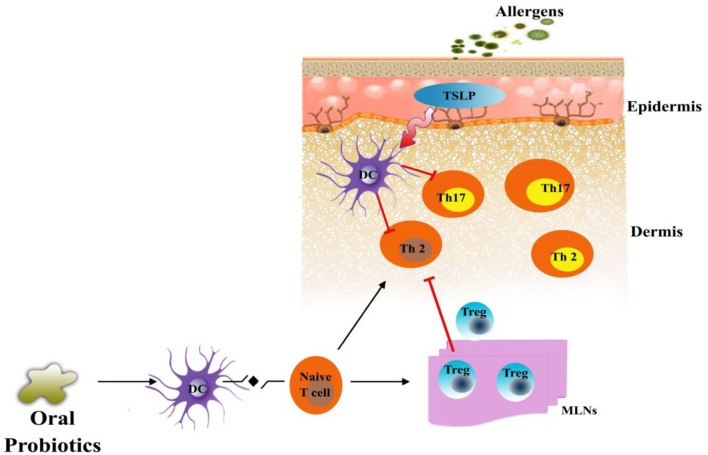
**Proposed mechanism of probiotics in an animal model of AD.** Exposure of atopic skin to a potential allergen enhances the expression of thymic stromal lymphopoietin (TSLP) that is known to activate dendritic cells (DC). Stimulated dendritic cell direct differentiation of naïve T-cell into Th2 cells and Th17 cells which, are known as the mediators of allergic inflammation in skin. Probiotics could inhibit the allergic inflammation by increasing the population of regulatory T cells (Tregs) in the mesenteric lymph nodes of patients. These Tregs could migrate to the site of inflammation and suppress the Th2 and Th17 mediated allergic response or directly reduce the expression of TSLP.

The discovery of Th17 cells as the mediators of allergic inflammation in a mouse model of asthama superseded the previous modes of action of probiotics against allergic diseases (Feleszko et al., 2007). The application of probiotics reduced the inflammation by suppressing the levels of INF-γ, IL-4, and Th17 cells in splenic CD_4_ T-cells and increasing the expression of IL-10 and Treg-related cytokines in mesenteric lymph nodes as shown in **Figure 1** (Jan et al., 2012). Probiotics also impose an inhibitory effect on the maturation of dendritic cells and, therefore, inhibit naive T-cells from differentiating into Th2 cells, which triggers inflammation in the skin (Kwon et al., 2010; Weiss et al., 2011). The differentiation of naïve T cells mediated by dendritic cells are known to be regulated by thymic stromal lymphopoietin (TSLP), a process that could be inhibited by probiotics (Weiss et al., 2011). Kim et al. (2013a,b) confirms the involvement of dendritic cells, following the transfer of mature dendritic cells in mice, in the suppression of allergic disease by probiotics.

## Conclusion

Probiotics for the prevention or intervention of AD is a vast underestimated area of research; and as a result, there is no reliable evidence to date that strongly supports their safe application. In spite of the weak evidence, a considerable number of clinicians prescribe the use of probiotics for the prevention of eczema. The regular instillation of probiotics in daily use at an early age could help in preventing the initiation of eczema. However, several variables, such as the use of antibiotics, prenatal and postnatal diet, mode of delivery, and surrounding allergenic environment in the home, could impact the early-life colonization of probiotic strains. Nevertheless, the clinical administration of probiotics may become more widespread if the remaining questions are answered with strong evidence: what type of probiotic strain should be used? What dosage and time of administration should be used? At what time of life is the use of probiotics more efficacious? And most importantly, should the use of probiotics be personalized? Current analysis of the role of probiotics in the prevention of AD reveals that a positive effect may be related to the type of probiotic strain used, the method of administration, onset time, as well as the dose size and duration of treatment. However, these uncertainties need to be further clarified before corroborating the preventive impact of probiotics in the prevention and/ or treatment of AD.

## Author Contributions

IR and VB designed and wrote the manuscript, JL and WP conception and design the outline, SK and YK did the critical review and approved the manuscript.

## Conflict of Interest Statement

The authors declare that the research was conducted in the absence of any commercial or financial relationships that could be construed as a potential conflict of interest.
